# *KRAS* mutations in tumor tissue and plasma by different assays predict survival of patients with metastatic colorectal cancer

**DOI:** 10.1186/s13046-014-0104-7

**Published:** 2014-12-10

**Authors:** Jian-Ming Xu, Xiao-Jing Liu, Fei-Jiao Ge, Li Lin, Yan Wang, Manish R Sharma, Ze-Yuan Liu, Stefania Tommasi, Angelo Paradiso

**Affiliations:** Affiliated Hospital Cancer Center, Academy of Military Medical Sciences, Beijing, China; Department of Medicine, Section of Hematology/Oncology, University of Chicago, Chicago, IL USA; Affiliated Hospital Pharmacology Laboratory for Cancer Research, Academy of Military Medical Sciences, Beijing, China; National Cancer Research Centre, Istituto Tumori G Paolo II, Bari, Italy; Department of Gastrointestinal Oncology, Affiliated Hospital Cancer Center, Academy of Military Medical Sciences, No. 8 Dong Da Avenue, FengTai District Beijing, 100071 China

**Keywords:** Kras, Colorectal cancer, Prognosis

## Abstract

**Background:**

The optimal laboratory assay for detecting *KRAS* mutations in different biospecimens from patients with metastatic colorectal cancer (mCRC), and the clinical relevance of these gene alterations is still in question. We analyzed the prognostic–predictive relevance of *KRAS* status, determined in tumor and plasma DNA by two different assays, in a large mono-institutional series of mCRC patients.

**Methods:**

DNA sequencing and peptide-nucleic-acid-mediated-polymerase chain reaction clamping (PNA-PCR) were used to determine *KRAS* status in 416 tumor and 242 matched plasma DNA samples from mCRC patients who received chemotherapy only. Relationships with outcomes were analyzed with respect to the different assays and tissue types.

**Results:**

PNA-PCR was significantly more sensitive in detecting *KRAS* mutations than sequencing (41% vs. 30%, p < 0.001). *KRAS* mutations were more frequent in tumor tissue than in plasma (sequencing, 38% vs. 17%, p < 0.001; PNA-PCR, 47% vs. 31%, p < 0.001). Median OS was consistently shorter in *KRAS*-mutated patients than *KRAS* wild-type patients, independent from the assay and tissue tested; the largest difference was in plasma samples analyzed by PNA-PCR (*KRAS* mutated vs. wild-type: 15.7 vs. 19.1 months, p = 0.009). No association was observed between *KRAS* status and other outcomes. When tumor and plasma results were considered together, median OS in patients categorized as tissue/plasma *KRAS* negative/negative, tissue/plasma *KRAS* discordant, and tissue/plasma *KRAS* positive/positive were 21.0, 16.9 and 15.4 months, respectively (p = 0.008).

**Conclusions:**

*KRAS* mutation status is of prognostic relevance in patients with mCRC. *KRAS* mutations in both tumor tissue and plasma are a strong prognostic marker for poor outcomes.

**Electronic supplementary material:**

The online version of this article (doi:10.1186/s13046-014-0104-7) contains supplementary material, which is available to authorized users.

## Background

K-RAS is a key oncogene member of the mitogen-activated protein kinase signaling pathway [1,2]. Research primarily in colorectal cancer shows that *KRAS* mutations occur most commonly in codons 12 and 13, usually precede the development of malignancy [3,4], and are maintained in secondary disease sites [5].

Several studies have addressed the prognostic-predictive value of *KRAS* mutational status in colorectal cancer patients [6]. It is now well established that *KRAS* mutations are the main reason for resistance to anti-epidermal growth factor receptor (EGFR) antibodies [7,8], and account for nearly two-thirds of EGFR downstream effector alterations in colorectal cancer [9]. *KRAS* mutations are also a predictive factor for response to tyrosine kinase inhibitors [10].

Clinical data regarding the prognostic value of *KRAS* mutations in patients with metastatic colorectal cancer (mCRC) treated with chemotherapy remain inconclusive [1]. Most studies addressing this question were retrospective or included limited patient numbers [6]. Some prospective studies also reached discordant conclusions [11] because the data were derived from the control arm of randomized trials of anti-EGFR antibodies as first-line treatment in combination with chemotherapy. In these studies, cross-over to anti-EGFR therapy was permitted or patients received anti-EGFR therapy after study treatment, making prognostic interpretation difficult. The prognostic-predictive relevance of *KRAS* alterations to chemotherapy alone in mCRC has still to be determined in a large patient sample.

Although direct sequencing is widely accepted as the gold standard for mutation screening [12], this method requires that ≥25% of DNA alleles in the sample are mutated [6,13]. Over the past five years, several new techniques, including peptide-nucleic-acid-mediated polymerase chain reaction clamping (PNA-PCR), have emerged. These methods have higher sensitivity than direct sequencing, and permit the detection of lower mutation frequencies (1%-5%) [14-16].

Routine assessment of *KRAS* mutational status is generally performed in tumor samples and used to personalize treatment in patients with mCRC [14,17]. Oh et al. combined PNA-Mediated Asymmetric PCR with Melting Curve Analysis to detect several types of low-level KRAS mutations in colorectal cancer tissues [18]. However, a recent review of 11 clinical studies, reported that 29%-100% of patients presented with the same *KRAS* mutation in both blood and tumor samples suggesting that blood samples may also be suitable for determining *KRAS* status [19-21]. One study performed by Yu et al. has showed that PNA-PCR powered by pyrosequencing had the potenial to screen plasma KRAS mutations with high sensitivity and accuracy in pancreatic cancer patients [22].

We hypothesized that *KRAS* mutation status determined using PNA-PCR in tumor tissue and/or blood could be a powerful and easy-to-perform approach for planning treatment in patients with advanced colorectal cancer. In the present study, we analyzed the impact of *KRAS* status, determined by both direct sequencing and PNA-PCR methods in tumor and matched plasma samples, on clinical outcome in a large consecutive mono-institutional series of advanced colorectal cancer patients. All patients received oxaliplatin-based or irinotecan-based chemotherapy as first-line and second-line treatment, but never received biologic therapy.

## Methods

### Study design

Between January 2007 and June 2011, 566 consecutive patients with mCRC were admitted to the Affiliated Hospital Cancer Center of the Academy of Military Medical Sciences in Beijing and were treated with systemic chemotherapy. 416 patients meeting the inclusion criteria were enrolled into this study retrospectively. The study was approved by the ethical committee of Affiliated Hospital, Academy of Military Medical Sciences (ID-2011-91). All patients provide their written informed consent to participate in this study.

The inclusion criteria were: completion of ≥2 cycles of oxaliplatin-based or irinotecan-based chemotherapy as first-line treatment; no anti-EGFR or anti-vascular endothelial growth factor (VEGF) treatment; measurable disease; adequate follow-up for disease and survival assessment; adequate tumor tissue and paired plasma samples (if available) taken before chemotherapy; tumor specimens with ≥50% tumor cells confirmed by certified pathologists; and tumor specimens obtained from surgical resection, colonoscopy biopsy, or metastatic site biopsy. Written informed consent was obtained from each patient.

Reasons for excluding patients (n = 150) from the consecutive series were: subsequent treatment with anti-EGFR and/or anti-VEGF antibodies (n = 85); non-measurable disease (n = 23); received only 1 chemotherapy cycle (n = 11); inadequate follow-up (n = 9); tumor tissue unavailable (n = 15); <50% tumor cells confirmed by pathologists (n = 4); and inadequate tumor specimen (n = 3).

### Tissue samples

Formalin-fixed, paraffin-embedded (FFPE) tumor tissues were retrieved at room temperature, assessed by sectioning, and hematoxylin-eosin stained by certified pathologists. The pathologist was responsible for tissue block selection and evaluation of neoplastic cellularity. Each evaluation was confirmed by two pathologists.

Whole blood samples were collected for DNA extraction from each participant before any invasive procedures or therapy. Five milliliters of peripheral blood were collected from each participant in a vacutainer system with lithium-heparin. Plasma was immediately separated from the cellular fraction by centrifugation at 1,500 × *g* for 10 min and, after aliquotation, frozen at −80°C.

Direct sequencing and PNA-PCR were performed with tumor and plasma samples to determine *KRAS* status.

### DNA extraction and *KRAS* mutation analysis

Genomic DNA of tumor tissue was extracted using EZNA™ FFPE DNA Kit D3399 (Omega Bio-Tek, Inc, Norcross, GA, USA). Free plasma was purified by NucleoSpin® Plasma, N.740900 (Macherey-Nagel GmBH & Co, Düren, Germany). All DNA samples were stored at −80°C. Since most *KRAS* mutations occur at codons 12 or 13 of exon 2, PCR primers were designed to amplify the corresponding region. PCR products were purified by EZNA® Cycle Pure Kit (Omega Bio-Tek, Inc, Norcross, GA, USA).

Direct sequencing of KRAS in plasma or FFPE was performed with Applied Biosystems® 3100 Genetic Analyzer (Life Technologies, Carlsbad, CA, USA) according to manufacturer’s protocol.

All available tumor and plasma samples were tested by PNA-PCR. Peptide-nucleic-acid (PNA) oligomers are non-extendable oligonucleotides. In PNA-mediated PCR clamping, PNA oligomers suppress the amplification of the complementary sequence because PNA are not substrates for DNA polymerase. The PNA oligomers (with sequence of CTACGCCACCAGCTC) covered wild-type codons 12 and 13 of *KRAS*. The nested PCR system included: 50 ng genomic DNA; 25 μL MightyAmp Buffer; 1 μL MightyAmp DNA Polymerase (DR071, TaKaRa Bio Inc, Shiga, Japan); 0.01 μM *KRAS* primers; and 0.2 μM PNA. After initial denaturation at 94°C for 5 min, PCR amplification consisted of 35 cycles: 94°C for 30 s; 70°C for 10 s; 56°C for 30 s; and 68°C for 30 s. This was followed by elongation at 68°C for 5 min with final cooling at 4°C. The sequences of primer pairs in the first run were GTGTGACATGTTCTAATATAGTCA and GAATGGTCCTGCACCAGTAA, while the inner forward primer ATGTTCTAATATAGTCACATTTTC and reverse primer GGTCCTGCACCAGTAATATGCA were used in the second run. The KRAS mutations in the nested PCR products were detected by subsequent Sanger sequencing.

Personnel responsible for mutation analysis were blinded to clinical outcomes. All experiments were performed at the Affiliated Hospital Pharmacology Laboratory for Cancer Research, and all the samples were genotyped twice for quality control purposes.

### Statistical analysis

The chi-squared test was used to determine the association between baseline characteristics, clinical response and *KRAS* status. Kaplan-Meier methods were used to estimate progression-free survival (PFS, defined as time between first day of first-line chemotherapy and disease progression or death from any cause) and overall survival (OS, defined as time between first day of first-line chemotherapy and death from any cause or date of last follow-up). Response was evaluated by the Response Evaluation Criteria in Solid Tumors. Cox proportional hazards models were used for multivariate analyses of baseline characteristics and *KRAS* mutations related to OS. In the multivariate Cox proportional hazard regression model, gender, age, Eastern Cooperative Oncology Group (ECOG) performance status, metastatic site, and *KRAS* status were used as covariates. The association between *KRAS* status in tumor tissue and plasma free DNA was examined by the Kappa test. All statistical tests were performed using SAS 9·2 software (SAS Institute, Cary NC, USA).

## Results

### Patient characteristics

A total of 416 patients met the enrollment criteria and had primary or metastatic tumor samples; 242 patients had paired plasma DNA samples. The characteristics of the patients were: 218 men and 198 women; median age of 56 (range, 26–87) years; and 365 patients (87.7%) had an ECOG performance status of 0–1. The characteristics of the study population are shown in Additional file 1: Table S1.

### *KRAS* mutation analysis

In order to verify the accuracy of the assays in detecting *KRAS* mutation status, 658 samples (416 primary tumors; 242 plasma samples) were analyzed by both direct sequencing and PNA-PCR (Table 1). Overall, PNA-PCR identified a higher percentage of *KRAS* mutated cases than direct sequencing (41% and 30%, respectively; p < 0.001). This difference was evident in tumor tissue (47% and 38%, respectively; p < 10^−8^) and plasma samples (31% and 17%, respectively; p < 10^−8^). PNA-PCR was able to detect *KRAS* mutations in an additional 39 tumor and 35 plasma samples where direct sequencing failed (Table 1). There were no cases in which *KRAS* mutations were detected by direct sequencing but not PNA-PCR. When *KRAS* mutations were further characterized by location (i.e. codons 12 or 13), the sensitivity of PNA-PCR was consistently higher (Table 1).

**Table 1:** ***KRAS*** **status analyzed in tumor tissue and plasma samples by direct sequencing and PNA-PCR assays**

		**Tumor tissue**	**Plasma**	**Total**
**Assay**	***KRAS*** **status**	**(n = 416)**	**(n = 242)**	**(n = 658)**
	Wild type	260 (63)	201 (83)	461 (70)
	Mutated	156 (38)	41 (17)	197^*^ (30)
	Codon 12	118 (28)	30 (12)	148 (22)
Direct sequencing	12 D	50	13	63
	12 V	39	10	49
	Other	29	7	36
	Codon 13	38 (9)	11 (5)	49 (8)
	Wild type	221 (53)	166 (69)	387 (59)
	Mutated	195 (47)	76 (31)	271^*^ (41)
	Codon 12	133 (32)	56 (23)	189 (29)
PNA-PCR	12 D	57	26	83
	12 V	46	17	63
	Other	30	13	43
	Codon 13	62 (15)	20 (8)	82 (12)

Data expressed as n (%).

*p < 0.001.PNA-PCR, peptide-nucleic-acid-mediated polymerase chain reaction clamping.

### *KRAS* mutations in tumor tissue and plasma

In order to ascertain whether plasma *KRAS* analysis is predictive of tumor *KRAS* mutation status, we assessed DNA samples from both tumor tissue and pre-surgery plasma of 242 patients (Table 2). Overall, *KRAS* mutations were more frequent in tumor than in plasma samples, and this was independent of the assay; *KRAS* mutation rates in tissue versus plasma were 38% vs. 17% with direct sequencing (p < 0.001) and 47% vs. 31.4% with PNA-PCR (p < 0.001) (Table 1). Only a small percentage of patients showed *KRAS* mutations in plasma but not in tumor tissue (5% by either method) (Table 2). The higher frequency of *KRAS* mutations in tumor tissue was also evident when different *KRAS* mutation subgroups were considered (Table 1).

**Table 2:** **Comparison of** ***KRAS*** status in tumor tissue and pre-surgery plasma in 242 patients with available paired samples*

	**Tumor tissue**				
**Plasma**	***Direct sequencing***				
	***Wild type***	***Mutated***	***Total***	***Kappa***	***p value*** ^***†***^
***Direct sequencing***					
Wild-type (%)	138 (57)	63 (26)	201	·278	<0 · 001
Mutated (%)	11 (5)	30 (12)	41		
Total	149	93	242		
*PNA-PCR*	*PNA-PCR*				
Wild-Type (%)	113 (47)	53 (22)	166	·456	<0 · 001
Mutated (%)	12 (5)	64 (26)	76		
Total	125	117	242		

*Kappa test was used to estimate the concordance of *KRAS* status between tumor tissue and plasma samples.

^†^All Wald statistical tests were two-sided.

PNA-PCR, peptide-nucleic-acid-mediated polymerase chain reaction clamping.

### Clinical outcomes and prognostic analysis

The median follow-up duration (calculated from the first day of first-line chemotherapy) was 26.9 (range, 10.0-62.5) months. Overall, 125 patients (30.0%) achieved a partial response. The median PFS of all patients was 6.1 (95% confidence intervals [CI], 4.8-7.5) months and the median OS was 17.4 (95% CI, 16.0-18.8) months.

We retrospectively analyzed the association between *KRAS* status and patient characteristics. There were no significant differences between gender, ECOG, performance status (0–1 vs. 2), metastatic site, and second-line treatment in patients with *KRAS* wild-type vs. *KRAS* mutant samples. We also analyzed the association between *KRAS* status and clinical outcomes following first-line chemotherapy (Table 3).

**Table 3:** **Prognostic value of** ***KRAS*** **status analyzed in tumor tissue and plasma by different laboratory assays in colorectal cancer patients**

			**Prognostic value**					
**Tissue and testing method**			**PFS**			**OS**		
	***KRAS*** ** status**		**Median PFS (mo)**	**95% Cl**	***P*** **value** ^*^	**Median OS (mo)**	**95% Cl**	***P*** **value** ^*^
*Tumor*								
Sequencing	Wild-type	260	6·1	4·5-7·7	·473	18·3	16·2-20·4	·064
	Mutated	156	5·8	3·4-8·2		15·9	14·4-17·4	
PNA-PCR	Wild-type	221	6·2	4·5-7·9	·360	19·5	17·2-21·8	·025
	Mutated	195	5·9	4·0-7·8		16·9	15·6-18·2	
*Plasma*								
Sequencing	Wild-type	201	6·1	5·5-6·6	·489	18·3	15·9-20·7	·037
	Mutated	41	5·4	4·9-5·8		15·7	9·3-22·1	
PNA-PCR	Wild-type	166	6·1	5·5-6·7	·274	19·1	16·8-21·4	·009
	Mutated	76	5·7	5·3-6·1		15·7	13·0-18·4	

*Log-rank test. All statistical tests were two-sided.

CI, confidence interval; mo, month; OS, overall survival; PFS, progression-free survival; PNA-PCR, peptide-nucleic-acid-mediated polymerase chain reaction clamping.

#### Tumor DNA analysis

**Figure 1:**
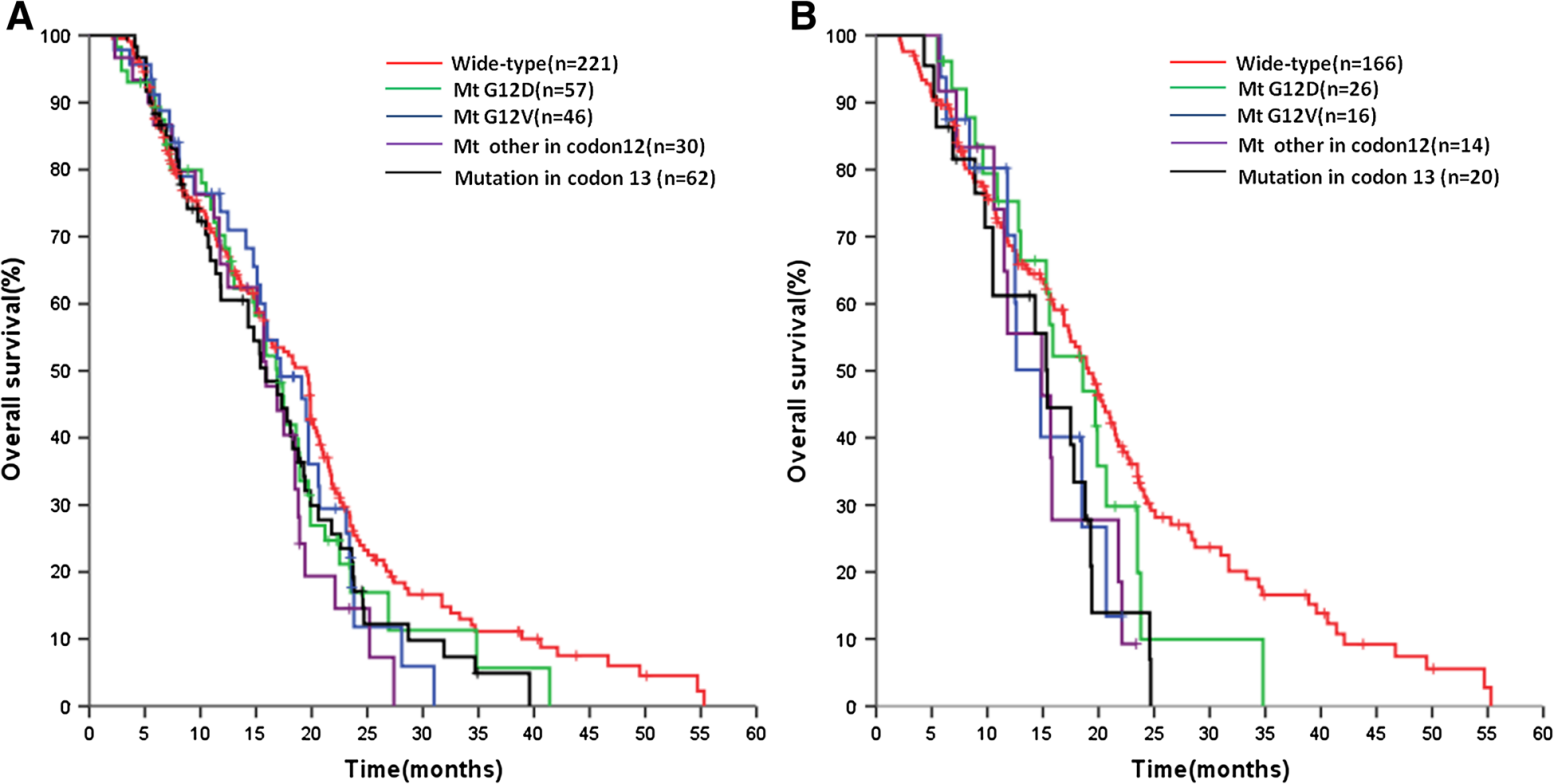
**Overall survival by** ***KRAS*** **mutation status in tumor tissue (A) and plasma (B) samples detected by PNA-PCR (see text for further details).**

There was no significant relationship between clinical response in patients with and without *KRAS* mutations; this was also true when subsets of patients carrying specific alterations in codons 12 or 13 were analyzed separately (data not shown). No significant difference between PFS in patients with *KRAS* wild-type vs. *KRAS* mutant samples was evident, regardless of whether mutation status was determined by direct sequencing (6.1 vs. 5.8 months; p = 0.473) or PNA-PCR (6.2 vs. 5.9 months; p = 0.360) (Table 3). However, OS was prolonged in patients with *KRAS* wild-type vs. *KRAS* mutant tumor samples determined by DNA sequencing (18.3 vs. 15.9 months; p = 0.064), and was statistically significant when mutation status was determined by PNA-PCR (19.5 vs. 16.9 months; p = 0.025). Figure 1A shows OS curves according to *KRAS* mutation status and by codon in tumor; the worst prognosis was evident in the subgroup of patients carrying mutations different from V and D in codon 12.

The multivariate analysis showed that frequency and type of *KRAS* mutations was the only covariate, other than ECOG performance status, that were significantly associated with OS (ECOG performance status: odds ratio [OR] 3.03, 95% CI 2.21-3.41, p < 0.001; *KRAS* wild-type vs. mutated: OR 1.37, 95% CI 1.06-1.70, p < 0.008). This association was independent of the assay but stronger with PNA-PCR.

#### Plasma DNA analysis

No relationship between *KRAS* status in plasma DNA samples and clinical response (data not shown) or PFS was observed (Table 3). Median OS was significantly longer in patients with *KRAS* wild-type vs. *KRAS* mutant samples, in particular when mutation status was determined by PNA-PCR (19.1 vs. 15.7 months; p = 0.009). Figure 1B shows OS curves according to *KRAS* mutation status and by codon in plasma.

The multivariate analysis demonstrated that the frequency and type of *KRAS* mutations was the only covariate, other than ECOG performance status, significantly associated with OS (ECOG performance status: OR 3.06, 95% CI 2.22-3.46, p < 0.001; *KRAS* wild-type vs. mutated: OR 1.35, 95% CI 1.05-1.65, p < 0.008). This association was independent of the laboratory assay but was stronger for PNA-PCR.

#### Combined analysis of DNA from tumor and plasma

For the analysis of the prognostic impact of *KRAS* mutation status, we used data from patients for which DNA from both tumor tissue and plasma were available (n = 242). Each patient was classified as *KRAS* positive (i.e. *KRAS* mutations in tumor and plasma samples), KRAS negative (i.e. *KRAS* wild-type in tumor and plasma samples), or with discordant *KRAS* status in tumor and plasma samples. This analysis was performed using the results obtained with PNA-PCR only.

**Figure 2:**
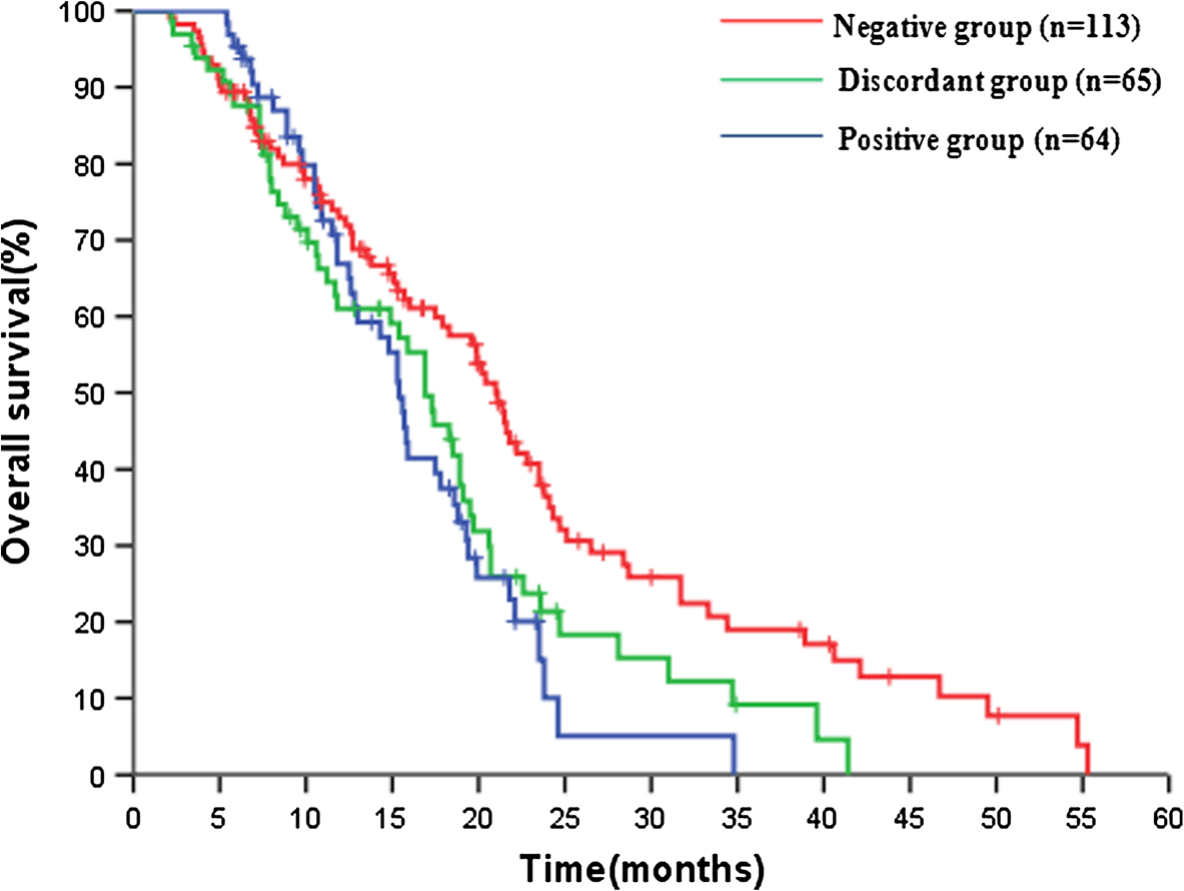
**Prognostic value of** ***KRAS*** **status analysed either in tumor tissue or plasma samples by PNA-PCR.** According to analyses of tumor tissue and plasma, *KRAS* status of each patient was categorized as: negative (KRAS wild type in tumor and matched plasma samples), discordant, (*KRAS* status discordant in tumor and matched plasma samples); positive (*KRAS* mutated either in tumor tissue and matched plasma samples.

While there was no relationship between *KRAS* status and clinical response or PFS, the analysis of OS showed a statistically significant association between the three cohorts (Table 4 and Figure 2). This association was confirmed in a multivariate analysis (Additional file 2: Table S2).

**Table 4:** **Prognostic value of combined tumor/plasma** ***KRAS*** **status analyzed by PNA-PCR in a series of 242 metastatic colorectal cancer patients with matched samples**

***KRAS*** status*		**Median OS**		
**Tumor/Plasma**	**No.**	**(mo)**	**95% Cl**	***P*** **value**
Negative/Negative	113	21.0	19.226-22.774	0.008
Discordant	65	16.9	14.184-19.616	
Positive/Positive	64	15.4	14.270-16.530	

*KRAS status was categorized as: negative, mutation-negative tumor and matched plasma samples; discordant, discordant *KRAS* status in tumor and matched plasma samples; positive, mutation-positive tumor tissue and matched plasma samples.

CI, confidence interval; mo, month; OS, overall survival; PNA-PCR, peptide-nucleic-acid-mediated polymerase chain reaction clamping.

## Discussion

In spite of the clear role that *KRAS* alterations play in the pathogenesis and progression of colorectal cancer, several aspects concerning their clinical validation remain inconclusive or are not yet fully explored [1]. Among them, the role of more sensitive laboratory assays for *KRAS* mutation analysis, the possibility of using circulating DNA to acquire information on *KRAS* status, and the possible prognostic-predictive value of these assays. The present study addresses all of these questions in a large mono-institutional series of patients for whom colorectal cancer tissue and plasma samples were available.

PNA-PCR has been reported to have a higher sensitivity than direct sequencing, permitting the detection of mutations at frequencies as low as 1%-5% [23]. We performed 658 assays by PNA-PCR and direct sequencing and found a significantly higher percentage of *KRAS* gene alterations with PNA-PCR (41% vs. 30%; p < 0.001), which was independent of the sample source (FFPE tumor samples or frozen plasma). A similar result has been reported in other studies [24,25] . Further, there were no cases in our study in which mutations were detected by direct sequencing but not PNA-PCR. We suggest that PNA-PCR is more accurate than direct sequencing and may be a clinically useful assay.

It has been suggested that circulating DNA is a suitable and reliable source of DNA for tumor somatic gene alterations [26] , but is this the case for the *KRAS* gene? In our study, we observed *KRAS* mutation detection rates in plasma samples of 17% and 31% by direct sequencing and PNA-PCR, respectively. Recent research indicates that the mutation detection rates in tumor tissue and plasma are influenced by disease stage [27,28], metastatic sites and performance status, since the major source of plasma DNA is from necrotic or apoptotic tumor cells [26]. Therefore, there would be a greater likelihood of detecting *KRAS* mutations in patients with a larger tumor burden or after multiple lines of therapy. Our study population was chemotherapy-naïve, which would be expected to result in a lower detection rate in plasma DNA.

A recent literature review [15] confirmed that *KRAS* alterations in ctDNA from plasma are significantly predictive of *KRAS* tumor status with a concordance between tumor and plasma status ranging from 29% to 100%. We analyzed 242 paired tumor and plasma samples, the largest series reported to date, and showed that determining *KRAS* status in plasma is feasible and provides information on gene status concordant with tumor DNA analysis by PNA-PCR in 73% of cases (Table 2). Although we showed that PNA-PCR can detect more *KRAS* mutated cases and that the discrepancy might be due to cases showing a mutation in the primary tumor and not in plasma, and this fact is in agreement with other recent studies which addressed the same question [29,30], further optimization of this methods also might increase the concordance between tumor and plasma as the investigation of KRAS and BRAF mutations consistence in tumor tissue and cfDNA [31]. We also found that 5% of cases that are mutant in plasma but wide-type in the tissue with both methods. The reasonable explanation for this is the heterogenity of cancer tissue, which means that the sections used in the detection only represented part of the tumor and did not contain the DNA carrying KRAS mutations that released into blood from other parts of tumor. Our study indicates that, if a tumor sample is unavailable or insufficient, using a more sensitive method than PNA-PCR assay such as ultra deep sequencing technology on a plasma DNA sample might be an alternative way to determine *KRAS* status in metastatic colorectal cancer.

The prognostic-predictive value of *KRAS* mutations in mCRC remains controversial. Recently, Ren et al. [6] did a systematic review and meta-analysis on this topic and identified 13 studies which showed that *KRAS* mutations are significantly associated with OS, but no separate analysis for studies in metastatic disease was performed. Yokota [32] also did not consider mCRC separately. However, Loriot [1] reviewed 6 clinical studies specifically looking at *KRAS* status in stage IV colorectal cancer and reported that *KRAS* mutations have no predictive value with conventional chemotherapy. Our results confirm these observations. *KRAS* status, and *KRAS* mutations in codons 12 or 13, had no predictive relevance for clinical response to chemotherapy or PFS. This finding was independent of assay utilized and DNA source. The conclusions were different when OS was considered. OS was influenced by *KRAS* status analyzed by PNA-PCR in plasma free DNA (univariate analysis, 15.7 months for *KRAS* mutations vs. 19.1 months for *KRAS* wild-type; p < 0.009; multivariate analysis, OR 1.35, 95% CI 1.05-1.65, p < 0.008). Interestingly, patients with *KRAS* mutations, regardless of their location, had a poorer prognosis than *KRAS* wild-type patients (Figure 1), although the differences between them were not significantly different. Overall, our results seem to confirm that *KRAS* mutations are associated with a more aggressive form of colorectal cancer.

It is difficult to compare our results with previous studies, as few studies have differentiated between *KRAS* mutations at codon 12 and 13. The largest study on this topic, RASCAL II which involved 3439 colorectal cancer patients, concluded that different gene mutations have different impacts on outcome, and in particular the codon 12 glycine to valine mutation, but it did not look specifically at stage IV disease [33]. Only one study [34] has considered advanced disease treated with systemic chemotherapy and showed that only the OS of patients carrying *KRAS* codon 13 mutations was significantly worse than for patients with wild-type *KRAS*. We further demonstrated that the impact of *KRAS* status on OS is independent from assay and DNA source, thus reinforcing the reliability of the evidence.

When patients were divided according to their combined plasma/tissue *KRAS* status, the best prognosis was observed in patients without mutations in either tumor or plasma, while the worst was evident in patients with mutations in both tissues. The subgroup with a discordant *KRAS* status showed an intermediate long-term prognosis. The prognostic relevance of this classification was confirmed in the multivariate analysis. The biological implications of this observation are unknown, but it seems possible that DNA from both primary tumor and plasma (which includes DNA from different sites) may provide additional clinically relevant information.

## Conclusion

This study showed that *KRAS* mutation status is able to predict long-term prognosis of patients with mCRC treated with conventional chemotherapy. This association was independent of the type of biospecimen and assay utilized for *KRAS* analysis, indicating that plasma DNA samples may provide an alternative biological source for *KRAS* mutation analysis. Further studies are required to corroborate this last hypothesis.

## Additional files

Additional file 1: Table S1. Baseline characteristics. Additional file 2: Table S2. Association of *KRAS* status with overall survival. Multivariate analysis with overall survival as dependent variable in a series of 242 advanced colorectal cancer patients; combined plasma/tumor *KRAS* status analyzed by PNA-PCR*.
