# Supplementary material for: KRAS mutations in tumor tissue and plasma by different assays predict survival of patients with metastatic colorectal cancer
Source: J Exp Clin Cancer Res. 2014 Dec 10;33(1):104. doi: 10.1186/s13046-014-0104-7 (PMC4272803; doi:10.1186/s13046-014-0104-7)
Supplement: Additional file 1: Table S1. — Baseline characteristics. [file 13046_2014_104_MOESM1_ESM.docx]

**Table S1.** Baseline characteristics

|  | **Total** |  |
| --- | --- | --- |
| **Characteristic** | **(N=416)** | **%** |
| Gender, n (%) |  |  |
| Female | 198 | 47·6 |
| Male | 218 | 52·4 |
| Age, years |  |  |
| Median | 56 |  |
| Range | 26-87 |  |
| ECOG performance status, n (%) |  |  |
| 0-1 | 365 | 87·7 |
| 2 | 51 | 21·3 |
| Metastatic site, n (%) |  |  |
| Liver | 127 | 30·5 |
| Lung | 99 | 23·8 |
| Liver and lung | 30 | 7·2 |
| Liver/lung and others | 45 | 10·8 |
| Other | 115 | 27·6 |
| Second-line treatment | 313 | 75·2 |

Percentages may be subject to rounding error.

ECOG, Eastern Cooperative Oncology Group.
