# Supplementary material for: KRAS mutations in tumor tissue and plasma by different assays predict survival of patients with metastatic colorectal cancer
Source: J Exp Clin Cancer Res. 2014 Dec 10;33(1):104. doi: 10.1186/s13046-014-0104-7 (PMC4272803; doi:10.1186/s13046-014-0104-7)
Supplement: Additional file 2: Table S2. — Association of KRAS status with overall survival. Multivariate analysis with overall survival as dependent variable in a series of 242 advanced colorectal cancer patients; combined plasma/tumor KRAS status analyzed by PNA-PCR*. [file 13046_2014_104_MOESM2_ESM.doc]

**Table S2.** Multivariate analysis with overall survival as dependent variable in a series of 242 advanced colorectal cancer patients; combined plasma/tumor *KRAS* status analyzed by PNA-PCR*

| **Characteristic** | **HR** | **95% Cl** | **p value**^†^ |
| --- | --- | --- | --- |
| ECOG performance status | 3.203 | 2.048-5.007 | <0.001 |
| Metastatic site | 1.102 | 0.995-1.221 | 0.063 |
| KRAS status^‡^ | 1.378 | 1.143-1.662 | 0.001 |

*Multivariate Cox proportional hazards regression analyses were used to estimate the adjusted hazard ratios for the baseline characteristics and overall survival of patients. Factors included gender, age, ECOG performance status, metastatic site and *KRAS* status in tissue and plasma detected by PNA-PCR.

^†^All statistical tests were two-sided.

^‡^ *KRAS* status was categorized as tissue/plasma negative/negative, tissue/plasma discordant, tissue/plasma positive/positive.

CI, confidence interval; ECOG, Eastern Cooperative Oncology Group; HR, hazard ratio; PNA-PCR, peptide-nucleic-acid-mediated polymerase chain reaction clamping.
